# Adhesion of two-dimensional titanium carbides (MXenes) and graphene to silicon

**DOI:** 10.1038/s41467-019-10982-8

**Published:** 2019-07-08

**Authors:** Yanxiao Li, Shuohan Huang, Congjie Wei, Chenglin Wu, Vadym N. Mochalin

**Affiliations:** 1 Department of Civil, Architectural, and Environmental Engineering, Missouri University of Science and Technology, Rolla, MO 65409 USA; 2 Department of Chemistry, Missouri University of Science and Technology, Rolla, MO 65409 USA; 3 Department of Materials Science and Engineering, Missouri University of Science and Technology, Rolla, MO 65409 USA

**Keywords:** Two-dimensional materials, Structural properties, Graphene

## Abstract

Two-dimensional transition metal carbides (MXenes) have attracted a great interest of the research community as a relatively recently discovered large class of materials with unique electronic and optical properties. Understanding of adhesion between MXenes and various substrates is critically important for MXene device fabrication and performance. We report results of direct atomic force microscopy (AFM) measurements of adhesion of two MXenes (Ti_3_C_2_T_x_ and Ti_2_CT_x_) with a SiO_2_ coated Si spherical tip. The Maugis-Dugdale theory was applied to convert the AFM measured adhesion force to adhesion energy, while taking into account surface roughness. The obtained adhesion energies were compared with those for mono-, bi-, and tri-layer graphene, as well as SiO_2_ substrates. The average adhesion energies for the MXenes are 0.90 ± 0.03 J m^−2^ and 0.40 ± 0.02 J m^−2^ for thicker Ti_3_C_2_T_x_ and thinner Ti_2_CT_x_, respectively, which is of the same order of magnitude as that between graphene and silica tip.

## Abstract (web summary)

The adhesion of two-dimensional transition metal carbides (MXenes) is important for potential MXene device fabrication and performance. Here, the authors show that adhesion of MXenes depends on their monolayer thickness and, in contrast to graphene, does not show number-of-monolayers dependency.

## Introduction

A large family of two-dimensional (2D) transition metal carbides and nitrides (MXenes) have emerged over the past few years as a class of materials with superior and highly tunable electronic and optical properties^1,2^. These properties render MXenes prospective materials for supercapacitors^3,4^, Li-ion and beyond Li-ion batteries^5,6^, triboelectric generators^7^, photonic diodes and Q-switched lasers^8^, sensors^9,10^, composites^11,12^, etc. More than 20 different MXenes have been synthesized by selective A element extraction with fluorine-containing etchants from bulk ternary compounds known as MAX phases^1,13^. MXenes have a general formula M_n+1_X_n_T_x_, where M represents an early transition metal (Ti, Zr, V, Nb, Ta, Cr, Mo, Sc, etc.), X is carbon or nitrogen, and n is an integer. T denotes the terminating functional groups (fluorine, hydroxyl, and other oxygen-containing groups) attached to MXene basal surfaces during synthesis^14–16^. Among experimentally available MXenes, Ti_3_C_2_T_x_ and Ti_2_CT_x_ are some of the most widely investigated. MXenes are intrinsically hydrophilic and yet, they have demonstrated higher electrical conductivity than solution processed graphene^17^. In addition, their exceptional electrochemical properties show great promise for flexible electronics and planar devices^18^. There are studies on MXene dispersions in organic solvents for incorporation into polymers, inks, etc^19^. As researchers move closer to device fabrication, adhesion to different materials, as well as other mechanical properties of MXenes become of greater importance. There is a handful of published data on the mechanical behavior of MXenes. Theoretical calculations using molecular dynamics (MD) have predicted the Young’s modulus of 597 GPa for bare Ti_2_C and 502 GPa for bare Ti_3_C_2_ MXenes^20^, whereas a recent experimental AFM indentation study of a single suspended Ti_3_C_2_T_x_ MXene flake reported the value of 333 ± 30 GPa^21^. Although no other experimental data for Ti_3_C_2_T_x_ or Ti_2_CT_x_ are available for comparison, the agreement between MD derived and experimental Young’s modulus is fairly good, taking into account the defective structure and surface terminations in real MXene samples that have not been captured in the simulations. MD modeling has also predicted a high bending rigidity of MXenes compared to single atomic layer 2D materials like graphene and hexagonal BN^22^, an important result for the development of sensing applications of 2D materials. However, to our knowledge, no theoretical or experimental studies of adhesive properties of MXenes have been reported to date.

Nanoindentation has been widely used to characterize adhesion of thin films^23–25^. In recent years, the adhesive interactions of monolayer and a few layer 2D materials have been intensively investigated. One of the important questions is to understand how the properties, in particular adhesive strength, change when transitioning from bulk to 2D form of a material. Answering this fundamental question will require systematic studies of the property (e.g., adhesive strength) while varying the thickness of a material without changing its atomic composition or type of bonding within the material. From this standpoint, the MXenes family represents another unique and still widely underutilized advantage, allowing to systematically change the thickness of the monolayer without changing the type of bonding or elemental composition of the material (e.g., going from Ti_2_C to Ti_3_C_2_ to Ti_4_C_3_, etc. within the same family of titanium carbide MXenes). Other 2D materials have fixed monolayer thickness and thus would not allow the systematic study of a property as a function of their monolayer thickness disentangled from other variables, such as elemental composition, chemical bonding, etc.

In this work, AFM was used to measure the adhesive properties of two MXenes (Ti_3_C_2_T_x_, Ti_2_CT_x_), as well as the mono-layer, bi-layer, and tri-layer graphene interacting with the oxidized silicon tip. The force-displacement responses were evaluated and compared. The measured adhesion force was converted into the adhesion energy using the Maugis-Dugdale^26^ theory and taking surface roughness into consideration. The experimentally measured adhesion energy between Ti_3_C_2_T_x_ and SiO_2_ is 0.90 ± 0.03 J m^−2^, which is higher than that of Ti_2_CT_x_ (0.40 ± 0.02 J m^−2^), thus the monolayer thickness was found to have a significant effect on the adhesion energy. However, in contrast to our experimental data for graphene, the number of MXene monolayers in a stacked MXene structure was found to have little impact on the adhesion energy.

## Results

### Surface profile

**Fig. 1:**
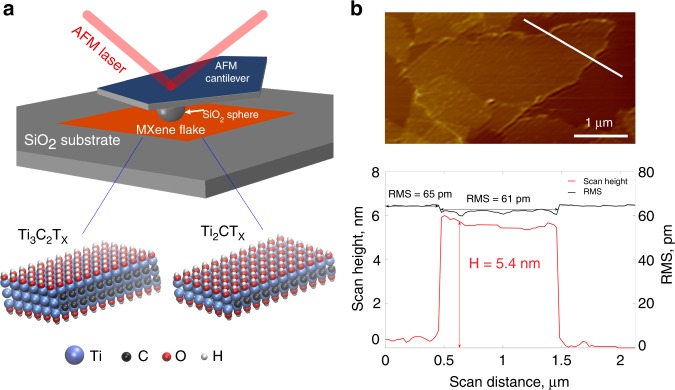
**a** Schematic representation of AFM indentation experiment, **b** AFM image and surface profile along the white line across the Ti_3_C_2_T_x_ flake deposited on SiO_2_ coated Si substrate

The surface roughness of the two types of MXene flakes was characterized using AFM scans in tapping mode with a 3 nm radius tip as shown in Fig. 1a. The RMS values for the MXene flake (Fig. 1b) are almost same as for SiO_2_ surface, indicating that MXene flakes are very thin and conform closely to the substrate.

**Fig. 2:**
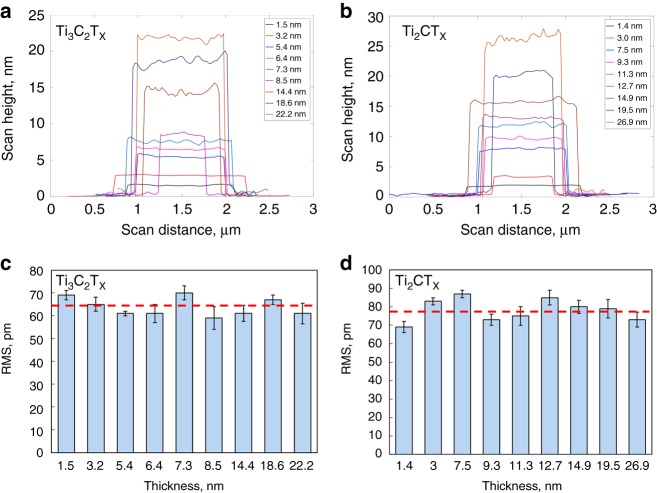
AFM line scans (**a**, **b**) and corresponding RMS values (**c**, **d**) for Ti_3_C_2_T_x_ and Ti_2_CT_x_ films of different thicknesses. Horizontal dash lines in bottom panels represent the average RMS for Ti_3_C_2_T_x_ and Ti_2_CT_x_, which were used in adhesion energy calculations. Error bars represent standard deviations in RMS values

Typical line scans for MXene films of different thicknesses are shown in Fig. 2a for Ti_3_C_2_T_x_ and Fig. 2b for Ti_2_CT_x_. The sharp changes in height at both sides of the line scans indicate the edges of the flakes. The horizontal length of the curves’ plateaus gives the average size of the MXene flakes between the two edges. The average RMS values for Ti_3_C_2_T_x_ and Ti_2_CT_x_ films (excluding the potentially oxidized edges) with different thicknesses were calculated and presented in Fig. 2c, d, respectively. Compared with the corresponding average thickness, the RMS values are relatively low (59–70 pm for Ti_3_C_2_T_x_, 69–87 pm for Ti_2_CT_x_) and within 5% of the corresponding thickness (compare top and bottom panels in Fig. 2), indicating relatively flat MXene surfaces. As follows from Fig. 2c, d, no clear thickness (i.e., the number of MXene monolayers) dependency was observed for the measured RMS values of the MXene films. The average RMS values were used in calculating the adhesion energies (below).

### Force-displacement response

**Fig. 3:**
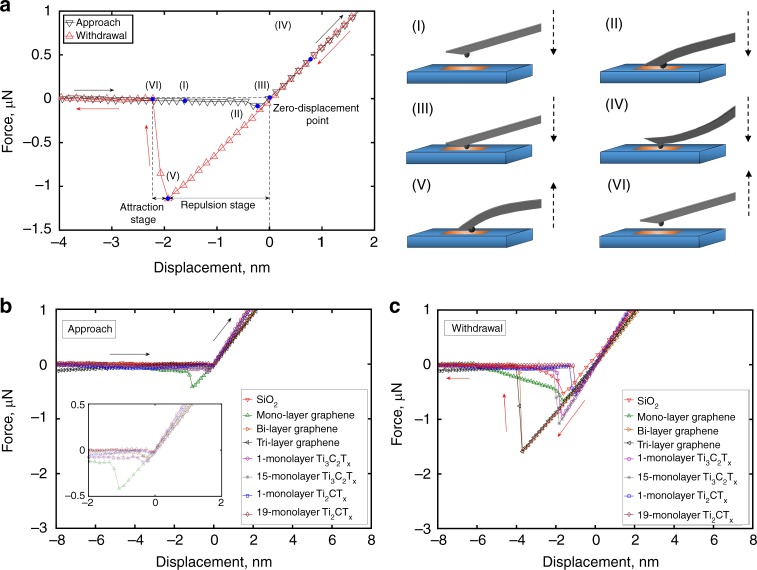
Force versus displacement graphs: **a** approach and withdrawal for 5-monolayer Ti_3_C_2_T_x_ sample and cartoons illustrating relative positions of the tip and the sample during different key stages of the AFM indentation process, **b** approach, **c** withdrawal for different samples

A typical force versus displacement response for tip approach and withdrawal measured on 5-monolayer Ti_3_C_2_T_x_ sample is shown in Fig. 3a. At the start of the experiment, the AFM tip was at a large distance from the sample surface. As the tip approached the sample surface (I) (indicated by the black line starting from the left side of the graph), a slowly increasing negative force (adhesion) was measured. At a certain displacement value (II), the “jump-in” phenomenon was observed resulting from sudden bending of the AFM cantilever towards the sample surface as the adhesion reached its local maximum. After this, the AFM cantilever bending reduced with the continued displacement of the cantilever towards the sample until there was no negative load on the cantilever (at this point the cantilever is not bent) and the tip was in firm contact with the surface—this corresponds to the zero-displacement point (III); from here the indentation process begins upon further increase of displacement (IV). During the indentation stage, the tip experienced a positive force on it since the cantilever bends out of the sample surface (compare cartoons IV and II).

In the withdrawal process starting from the rightmost part of the graph in Fig. 3a (indicated by the red line), the force on the tip reduced during the tip retraction. At 0 nm displacement (i.e., zero-displacement point), there is now a pulling force acting on the tip, which is balanced by its adhesion to the sample. When the displacement becomes slightly negative upon further withdrawal, the tip still adheres to the sample, resulting in cantilever bending towards the sample again (V). As the tip withdrawal continues, adhesion force approaches its maximum value before the tip “jumps-off” the sample surface (V to VI), turning the force acting on the cantilever into zero. The maximum adhesion force measured in point V (−1.11 µN in Fig. 3a) was then used to calculate the adhesion energy between the tip and sample surface.

For all samples, the adhesion forces measured during the tip approach (−0.02 to −0.42 µN, Fig. 3b) are generally lower than those recorded in the withdrawal stage (−0.5 to −1.56 µN, Fig. 3c). The maximum adhesion force measured between the tip and monolayer graphene is much lower than that for bi-layer or tri-layer graphene (Fig. 3c). However, in contrast to graphene adhesion, no number-of-monolayer dependency of the maximum adhesion force was found for the MXene films. The maximum values of adhesion force are higher for Ti_3_C_2_T_x_ (1.07 µN) than those for Ti_2_CT_x_ (0.53 µN). The “jump-off” (V to VI) observed for graphene, as well as MXene samples describes adhesion with a large interaction range, which may be a sign of the capillary force effect. Similar findings were reported in the nanoindentation experiments conducted on the surface of graphene^27^. The jump-off instability is worth mentioning at this conjunction as it might affect the accuracy of the measurements. However, this concern was eliminated by an accuracy analysis comparing the cantilever stiffness with the adhesion gradient during the experiment (Supplementary Note 1 and Supplementary Fig. 1). More force versus displacement data are provided in Supplementary Fig. 2. The average adhesion forces along with standard deviations for all samples are plotted in Supplementary Fig. 3a.

### Adhesion energy

**Fig. 4:**
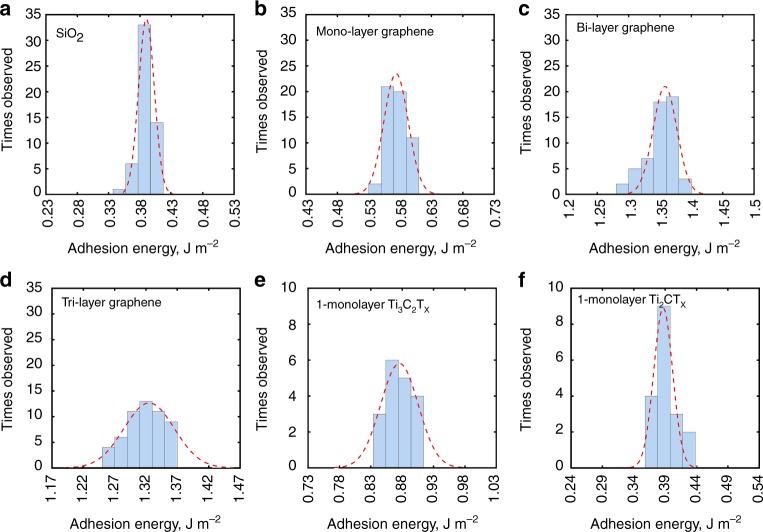
Histograms of calculated adhesion energies for: **a** SiO_2_/SiO_2_, **b** mono-layer graphene/SiO_2_, **c** bi-layer graphene/SiO_2_, **d** tri-layer graphene/SiO_2_, **e** 1-monolayer Ti_3_C_2_T_x_/SiO_2_, and **f** 1-monolayer Ti_2_CT_x_/SiO_2_. (The total count is 54 for SiO_2_ and graphene and 18 for MXene)

The adhesion energy was calculated from the measured “jump-off” (or maximum adhesion) force using the Maugis–Dugdale theory^28^,

1 $$W_{{\mathrm{adh}}} = \frac{{F_{{\mathrm{adh}}}}}{{{\mathrm{\lambda \pi }}R_{{\mathrm{tip}}}}}$$

where *W*_adh_ is the adhesion energy per unit area, *F*_adh_ is the maximum adhesion force measured during the withdrawal stage, *R*_tip_ is the tip radius and *λ* is an effective coefficient that depends on the model and data used ($\lambda = {\mathrm{1}}{\mathrm{.613}}$ for SiO_2_, $\lambda = {\mathrm{1}}{\mathrm{.587,}}\,{\mathrm{1}}{\mathrm{.543,}}\,{\mathrm{1}}{\mathrm{.543}}$ for mono-layer, bi-layer, and tri-layer graphene, correspondingly, $\lambda {\mathrm{ = 1}}{\mathrm{.560,}}\,{\mathrm{1}}{\mathrm{.558}}$ for 1-monolayer and 15-monolayer Ti_3_C_2_T_x_, and $\lambda {\mathrm{ = 1}}{\mathrm{.602,}}\,{\mathrm{1}}{\mathrm{.602}}$. for 1-monolayer and 19-monolayer Ti_2_CT_x_, correspondingly, see Supplementary Note 2). To account for the roughness effect, the tip roughness (RMS ≈ 180 pm provided by the vendor, Novascan, Inc.) and the average surface roughness of SiO_2_ (65 pm), graphene (58 pm, 60 pm and 63 pm for mono-layer, bi-layer, and tri-layer graphene, respectively) and MXenes (64 pm and 78 pm for Ti_3_C_2_T_x_ and Ti_2_CT_x_, rpectively), all shown in Supplementary Fig. 3b, were employed using the modified Rumpf model^28,29^. The actual adhesion energy is:

2 $$W_{{\mathrm{adh}}} = \left( {\frac{{F_{{\mathrm{adh}}}}}{{\lambda \pi R_{{\mathrm{tip}}}}}} \right)\left( {\frac{{\left( {{\mathrm{1 + }}\frac{{R_{{\mathrm{tip}}}}}{{{\mathrm{1}}{\mathrm{.48}}R_{{\mathrm{film}}}}}} \right)^{ - 1} + \left( {{\mathrm{1 + }}\frac{{{\mathrm{1}}{\mathrm{.48}}R_{{\mathrm{film}}}}}{{Z_0}}} \right)^{ - 2}}}{{\left( {{\mathrm{1 + }}\frac{{R_{{\mathrm{tip}}}}}{{{\mathrm{1}}{\mathrm{.48}}R_{{\mathrm{tip}} - {\mathrm{RMS}}}}}} \right)^{ - 1} + \left( {{\mathrm{1 + }}\frac{{{\mathrm{1}}{\mathrm{.48}}R_{{\mathrm{tip}} - {\mathrm{RMS}}}}}{{Z_0}}} \right)^{ - 2}}}} \right)$$

where *R*_film_ is the RMS value for the sample surface, $R_{{\mathrm{tip}} - {\mathrm{RMS}}}$ is the RMS value for the tip and *Z*_0_ is the equilibrium separation of two surfaces. In our experiments, the equilibrium separation *Z*_0_ is defined as zero-force distance between the surface of SiO_2_ tip and the sample surface, which was estimated to be 0.3 nm^30^. All adhesion energies in this work were calculated according to Eq. (2). The histograms of the adhesion energy between all sample surfaces including SiO_2_/SiO_2_, graphene/SiO_2_, Ti_3_C_2_T_x_/SiO_2_, and Ti_2_CT_x_/SiO_2_ are presented in Fig. 4. Gaussian fitting was applied to obtain the average adhesion energy and standard deviations for each specimen.

**Fig. 5:**
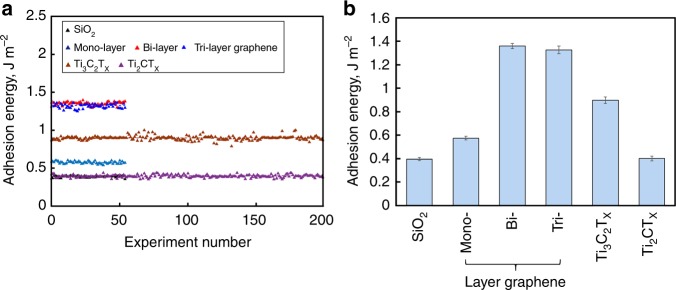
**a** Adhesion energy for all measured samples (since adhesion energy of Ti_3_C_2_T_x_ and Ti_2_CT_x_ thin films is independent of the film thickness, MXene films with different number of monolayers are represented by one symbol in the graph), **b** average adhesion energies with error bars indicating standard deviations

We compare all measurements and plot the average adhesion for each type of interactions in Fig. 5. The average adhesion energy for SiO_2_/SiO_2_ (0.40 ± 0.01 J m^−2^) is fairly close to the value obtained for piranha solution treated Si wafer by Na, et al. (0.33 ± 0.01 J m^−2^)^30^.

The adhesion energies of SiO_2_ with mono-layer, bi-layer, and tri-layer graphene are 0.58 ± 0.02, 1.36 ± 0.02, and 1.33 ± 0.03 J m^−2^, respectively, indicating the number-of-monolayer dependence. This experimental dependence shows that adhesion energy of graphene with SiO_2_ initially increases when going from mono-layer to bi-layer graphene and then stays constant when the number of graphene monolayers in the stack is increased to three. Similarly, a small increase in normal pull-off force that was more pronounced when going from 1 to 2 stacked graphene layers and less pronounced for a larger number of layers, has been predicted by modeling^31^. However, this trend was “not observed in experiments possibly due to the experimental uncertainty”^31^. Another relevant paper on the number-of-layer effect on adhesion of graphene^32^ mentions the opposite trend (measured by blister method), which also ceases when the number of graphene layers exceeds 2. There are also studies mentioning no number-of-layer effect on the pull-off force of graphene^33^. This broad spectrum of literature results may indicate the challenges associated with experimental measurements, as well as the differences between the measurement techniques (for example, the blister method inevitably measures the effect of shear force between the material and substrate along with adhesion), and differences in graphene sample preparation (mechanically exfoliated versus CVD graphene)^32^. However, for the purposes of our study it is important that: (i) modeling predicts a slight increase in the adhesion with a number of graphene monolayers^31^, while it is not easy to explain the trend in opposite direction^32^; and (ii) both modeling and experiments (even those where oppositely directed trend was observed) converge on that the number-of-layer trend for graphene ceases when this number exceeds 2–3, i.e., when the distance between the top-most and the bottom-most graphene layers is ~0.7–1 nm.

The average adhesion energy of the monolayer graphene measured in this work (0.58 ± 0.02 J m^−2^) is close to the results of Jiang and Zhu (0.46 ± 0.02 J m^−2^)^23^ and Torres et al. (0.57 J m^−2^)^34^. The small differences between these results are possibly due to different methods of graphene synthesis or different measurement methods. Jiang and Zhu measured graphene sample prepared by mechanical exfoliation, while CVD graphene was used by Torres et al. Here, we use an AFM experiment similar to Jiang and Zhu^23^, while Torres et al.^34^ measured using the nanoparticles method. Due to differences in sample preparation approaches, as well as adhesion energy measurement techniques, the authors could not provide explicit or definite reasons for the slightly higher adhesion measured in this work as compared to different literature data.

The average adhesion energy measured between SiO_2_ and 1-monolayer Ti_3_C_2_T_x_ is 0.90 ± 0.03 J m^−2^, which is about twice that between SiO_2_ and 1-monolayer Ti_2_CT_x_ (0.40 ± 0.02 J m^−2^), and larger than that between SiO_2_ and monolayer graphene. At the same time, the average adhesion energy between SiO_2_ and 1-monolayer Ti_2_CT_x_ is less than between SiO_2_ and monolayer graphene (Fig. 5). Since surface chemistry of Ti_3_C_2_T_x_ and Ti_2_CT_x_ is very similar as verified by the X-Ray photoelectron spectroscopy (XPS) (Supplementary Note 3 and Supplementary Fig. 4), we ascribe this result as mostly due to the monolayer thickness differences between the two MXenes (0.94 nm for Ti_3_C_2_T_x_ and 0.67 nm for Ti_2_CT_x_, respectively, calculated from X-Ray diffraction (XRD) data in Supplementary Fig. 5 and density functional theory optimized geometries in Supplementary Fig. 6). Although there was a clear monolayer thickness dependence of adhesion between each of the two MXenes and SiO_2_, we did not observe any number-of-monolayer (or sample thickness) dependency (Fig. 3b, c, Fig. 5a and Supplementary Fig. 2c, d). Details of the variation of measured adhesion energies among MXenes with different thickness and from different batches can be found in Supplementary Note 4, Supplementary Table 1, Supplementary Fig. 7, and Source Data. We hypothesize that the absence of the number-of-monolayer dependence for MXene stacks may be due to a large interlayer spacing (Supplementary Figs. 5, 6), which is several times more than the interlayer spacing in stacks of graphene. This large interlayer spacing in MXenes limits the experimentally measured adhesion by only the topmost (closest to the tip) layer contribution, preventing adhesive interactions between the underlying MXene layers and the tip. Referring to the above discussion on a number-of-monolayer dependence of adhesion for graphene, which ceases after 2–3 graphene monolayers in the stack (see also our experimental data in Fig. 5), we find that the distance at which this dependence ceases in case of stacked graphene is close to the thickness of either Ti_3_C_2_T_x_ or Ti_2_CT_x_ monolayers. It is, therefore, not surprising that no number-of-monolayer trend was observed here for the MXene samples, since their monolayer thickness and interlayer spacing are larger than those of graphene.

## Discussion

The adhesion energies between SiO_2_ coated Si tip and two types of MXenes were measured for the first time using AFM with a spherical tip and compared to adhesion energies between the same tip and mono-layer, bi-layer, and tri-layer graphene. The measured values are 0.90 ± 0.03 J m^−2^ and 0.40 ± 0.02 J m^−2^ for Ti_3_C_2_T_x_ and Ti_2_CT_x_, which are in the range of adhesion between SiO_2_ and graphene monolayer. A higher adhesion energy between SiO_2_ and Ti_3_C_2_T_x_ can be attributed to a thicker monolayer of this MXene compared to that of Ti_2_CT_x_. This observation provides the first illustration of how MXenes can be used to study the fundamental effects of monolayer thickness, disentangled from other factors, on mechanical properties of 2D materials. In contrast to graphene, no number-of-monolayers dependency was observed in this study for adhesion energy of multilayer MXene stacks, which we ascribe due to a larger interlayer spacing and monolayer thickness of the MXenes. The role of surface chemistry in adhesion interactions of MXenes with SiO_2_ and other substrates still remains unclear, but potentially it can be used to control the adhesion in the future, provided we could better understand MXene chemistry and tailor the functional groups terminating MXene surfaces.

## Methods

### Atomic force microscopy (AFM)

AFM (Digital Instruments Nanoscope IIIA) was conducted under the ambient conditions as illustrated in Fig. 1a. Thin films of graphene and MXene were mounted on the silicon substrate with a thin (~300 nm) layer of spontaneously formed silicon oxide. Surface profiles were measured using the tapping mode with a 3-nm radius silicon tip. The static eliminator (Static Sensing Ionizer, Keyence®) was used to neutralize any interfering electrostatic charges on the sample and/or AFM tip^35^. A 500-nm radius colloidal silicon tip with silicon dioxide (SiO_2_) surface was used to measure adhesive behavior of the samples. SEM images of the microsphere AFM tip are provided in Supplementary Fig. 8. The probe was calibrated before and after each experiment using the AFM grating to ensure that no damage to the probe occurred during the experiment. For each MXene flake, three measurements were conducted on the grid areas as illustrated in Supplementary Fig. 9a. As for graphene, nine measurements were performed on one flake due to a larger area of graphene flakes compared to MXene flakes. For further details and discussion of between the samples variations see Supplementary Note 4, Supplementary Table 2, and Source Data. The cantilever stiffness provided by the supplier (Novascan, Inc.) was ~2 N m^−1^. The force-displacement responses for the approach and withdrawal stages were recorded and analyzed.

The typical adhesive interaction of thin films involves the van der Waals, capillary, and electrostatic forces^36^. The capillary force is usually induced by water bridging the sample and tip surfaces^37,38^. We did check the effect of moisture by conducting AFM experiments with Ti_3_C_2_T_x_ while varying the relative humidity (RH, Supplementary Fig. 10). The maximum fluctuation of the adhesion force is within 5% of the average value for Ti_3_C_2_T_x_. These results show no significant humidity effect when RH is lower than 24%. We have also checked adhesion force versus time in air at room temperature to investigate the effect of sample oxidation. Supplementary Fig. 11a shows that the adhesion force stays constant within the first 48 h. The corresponding AFM images (Supplementary Fig. 11b) do not show any changes in the MXene surface morphology. After 48 h, the measured adhesion force drops dramatically accompanied by the visible changes in the AFM images, demonstrating signs of MXene oxidation (formation of TiO_2_ etc.). This indicates that the drop in adhesion force for Ti_3_C_2_T_x_ after 48 h exposure to ambient air is due to chemical changes of the MXene, emphasizing the need to work with freshly prepared MXene samples, which was always properly taken care of in this study: all MXene samples were measured within no more than 12 h from their preparation and drying.

### Synthesis of graphene and preparation of graphene samples on Si wafers

Large-area monolayer graphene was grown by chemical vapor deposition (CVD) on 2 × 10 cm copper foils (Alfa Aesar, CAS: 7440-50-8, LOT No. P17D009). During this process, gas species were fed into the reactor flow over the 25 µm thick piece of copper foil, where hydrocarbon precursors decomposed to carbon radicals at the copper surface and then formed monolayer graphene^39^.

To prepare the multilayer graphene, a copper foil with graphene on top was spin-coated with a layer of polymethyl methacrylate (PMMA) (3000 rpm for 30 s)^40^. The foil was then etched away in 0.2 mol L^−1^ FeCl_3_ and 0.2 mol L^−1^ (NH_4_)_2_S_2_O_8_ for 2 h^41^. The remaining graphene/PMMA was cleaned with deionized water, transferred onto freshly synthesized graphene on copper foil and heated at 50 °C in dry air to remove water. After another copper foil etching process, the graphene/graphene/PMMA sample was obtained. The tri-layer graphene was prepared by repeating this procedure. The graphene/PMMA, graphene/graphene/PMMA and graphene/graphene/graphene/PMMA samples were then transferred onto target Si (111)/SiO_2_ substrates and baked at 120 °C for 15 min. Prior to transfer, Si substrates were cleaned by 30 min bath sonication in acetone and hydrophilized in piranha solution (3 mL 30% H_2_O_2_ slowly added to 9 mL 98% H_2_SO_4_) for 24 h, followed by thorough rinsing with deionized water. Finally, PMMA was removed using acetone solution, yielding a sample of graphene on the Si/SiO_2_ substrate. The residue of polymer film was etched away at 400 °C in hydrogen^27^.

### Synthesis of MAX phases

Ti_3_AlC_2_ was prepared by pressureless synthesis method^42^. The initial powders of titanium (-325 mesh, 99%, Alfa Aesar), aluminum (-325 mesh, 99.5%, Alfa Aesar), and graphite (-325 mesh, 99%, Alfa Aesar) were ball-milled in 3:1.1:1.88 molar ratio in a polyethylene jar for 12 h at 100 rpm. Afterwards, the mixture was sintered at 1550 ^o^C for 2 h in Ar flow in an alumina boat using a tube furnace (GSL-1800X -KS60-UL, MTI Corporation). For Ti_2_AlC synthesis, the initial powders of titanium carbide (typically 2-micron size, 99.5%, Alfa Aesar), titanium (-325 mesh, 99%, Alfa Aesar), and aluminum (-325 mesh, 99.5%, Alfa Aesar) were ball-milled in a molar ratio of 0.85:1.15:1.05. The mixture was then heated at 1400 ^o^C for 4 h under Ar flow in an alumina boat. The resulting ceramics were manually crushed into powders using mortar and pestle.

### Preparation of MXene thin films on Si wafers

Ti_3_C_2_T_x_ MXene was synthesized by selective etching of Al from Ti_3_AlC_2_^43^. The etching was done by slowly mixing 0.3 g of Ti_3_AlC_2_ (-325 mesh, particle size ≤ 45 µm) to the etchant, prepared by dissolving 0.3 g LiF in 6 mL of 6 M HCl in a 50 mL plastic centrifuge tube. The mix was stirred for 24 h at room temperature, followed by repeated washing with deionized water and centrifugation until the pH of supernatant reached 5.5–6.0.

Ti_3_C_2_T_x_ aqueous colloidal solution was obtained via 5 min hand-shaking followed by 1 h centrifugation at 3500 rpm. Ti_3_C_2_T_x_ thin films on Si were prepared from the concentrated Ti_3_C_2_T_x_ colloidal solutions via interfacial film deposition method^8^. In order to prepare MXene thin films, 50–300 µL of Ti_3_C_2_T_x_ colloidal solution was mixed in 50 mL DI water together with 3–6 mL toluene added during 15 min of vigorous stirring. The dispersion was then poured directly into a beaker filled with 400 mL DI water and with a few pieces of pre-cleaned Si wafers placed at the bottom. After ~20 min standing still, the Ti_3_C_2_T_x_ film was self-assembled at the interface between water and toluene, and then the pieces of Si wafers were slowly lifted up from the solution through the interface, catching the interfacial MXene film. Finally, the MXene coated Si wafers were dried for 12 h in Ar flow at room temperature to avoid oxidation. Ti_2_CT_x_ colloidal solutions, as well as thin films were obtained using same methods, except the MAX phase in this case was Ti_2_AlC (-325 mesh, particle size ≤ 45 µm) and the MXene (Ti_2_CT_x_) film drying time was 4 h in Ar flow at room temperature.

By adjusting the concentration of MXene colloidal solution during sample preparation, MXene films with different thicknesses were deposited ranging between 1.4 to 26.9 nm. The adhesion measurements were carried out immediately after the films were dried.

### Moisture and oxidation effects

To investigate the moisture effect on adhesion measurements, three Ti_3_C_2_T_x_ samples after initial drying (12 h in dry Ar flow) were placed into a closed chamber with P_2_O_5_, where RH was monitored with CECOMINOD046940 monitor (Hyelec). The samples were removed for adhesion measurements when RH reached 24%, 21%, 18%, 15%, 12%, 9%, and 6% (Supplementary Fig. 10). To investigate the effect of oxidation on adhesion measurements, adhesion forces and AFM images were obtained for three Ti_3_C_2_T_x_ samples exposed to air for 24–96 h (Supplementary Fig. 11).

### Raman spectroscopy characterization

Raman spectroscopy was conducted using a Horiba LabRAM ARAMIS spectrometer with 632.8 nm laser. Supplementary Fig. 9b, c show Raman spectra of Ti_3_C_2_T_x_ and Ti_2_CT_x_ MXenes on Si and on cover glass. The peaks at 520 cm^−1^ recorded from MXenes deposited on Si wafer belong to Si. The MXene samples show characteristic Raman peaks of Ti_3_C_2_T_x_ at 206, 270, 374, 605, and 718 cm^−1^ ^44–46^, and of Ti_2_CT_x_ at 200, 300, 409, and 608 cm^−1^ ^47^, respectively. All the peaks originate from Ti–C bond vibrations that are also present in the Raman spectra of respective parent MAX phases published in literture^48^.

Graphene is usually characterized by G band at ~1600 cm^−1^ and 2D band at ~2650 cm^−1^. Raman spectra of our graphene transferred samples exhibited typical characteristics of high-quality graphene: for monolayer graphene the 2D/G intensity ratio is ~2.21 and a full-width at half maximum (FWHM) of 2D band is ~27 cm^−1^ ^49^. The 2D/G ratio and FWHM for the bi-layer graphene are 1 and 52 cm^−1^, and for the tri-layer graphene, these values are 0.5 and 63 cm^−1^ ^50^ (Supplementary Fig. 9d).

### X-Ray diffraction characterization

The structures of Ti_3_C_2_T_x_ and Ti_2_CT_x_ MXene thin films were characterized using X-Ray Diffraction (XRD, PANalytical, Phillips MPD) with Cu K < α > radiation ($\lambda \ {\mathrm{ = 1}}{\mathrm{.5406}}$ Å) at U = 45 kV, I = 40 mA.

### X-Ray photoelectron spectroscopy characterization

X-Ray photoelectron spectroscopy (XPS) measurements of Ti_3_C_2_T_x_ and Ti_2_CT_x_ MXene thin films were performed using a KRATOS AXIS 165 X-Ray photoelectron spectrometer with a monochromatic Al X-Ray source.

### Calculation of the number of MXene monolayers

The local number of MXene monolayers in our samples was calculated from a combination of AFM and XRD data as follows. The (002) peaks for Ti_3_C_2_T_x_ and Ti_2_CT_x_ are at $2\theta = 5.98^\circ$, and $6.48^\circ$, respectively (Supplementary Fig. 5). According to Bragg’s Law $2d{\mathrm{sin}}\theta = n\lambda$, the corresponding *d*-spacing values are 1.48 nm and 1.36 nm^51,52^, which are sums of the thickness of MXene monolayer and the interlayer spacing. Therefore, the number of MXene monolayers in different thin films can be calculated as the local AFM measured thickness of the films (Fig. 2) divided by the corresponding *d*-spacing values (Supplementary Fig. 6).

## Supplementary information

Supplementary Information

Source Data

## Data Availability

Data available on request from the authors.
