# Supplementary material for: Adhesion of two-dimensional titanium carbides (MXenes) and graphene to silicon
Source: Nat Commun. 2019 Jul 8;10:3014. doi: 10.1038/s41467-019-10982-8 (PMC6614499; doi:10.1038/s41467-019-10982-8)
Supplement: Supplementary file 1 — Supplementary Information [file 41467_2019_10982_MOESM1_ESM.pdf]

Supplementary information

Adhesion of Two-Dimensional Titanium Carbides (MXenes) and Graphene to Silicon

Li et al.

Supplementary Notes

Supplementary Note 1: Accuracy analysis of adhesion measurements

The jump-off instability has been the main concern when using AFM to measure adhesion forces. To avoid the unstable jump-off, the adhesive force gradient between the spherical tip and sample surface should be much greater than the spring stiffness of the AFM cantilever[1]. Here, we calculated the force *versus* displacement and obtained the force gradients for each type of specimen approaching the maximum adhesion. These values were compared with the slope of the cantilever's spring constant (2 N m^{-1}) to demonstrate the accuracy of the adopted method.

The pairwise interaction energy between the atom on the tip and the atom on the sample surface was assumed in the form of Lennard-Jones potential:

$$W_{\mathrm{LJ}}(r)=-\frac{C_1}{r^6}+\frac{C_2}{r^{12}} \text{. (1)}$$

Integration of Eq. (1) gives the interaction energy per unit area between the sample surface and the tip, E (see supporting information of reference 2)[2]:

$$E=-\gamma\left[\frac{3}{2}\left(\frac{Z_0}{Z}\right)^3-\frac{1}{2}\left(\frac{Z_0}{Z}\right)^9\right] \text{, (2)}$$

where Z_0 is the separation between tip and sample surface, Z is the distance between the sample surface and the spherical tip and γ is the adhesion energy per unit area at Z_0. The corresponding adhesive traction (f) and force gradient (k_{vdW}) can be obtained by taking the negative first and second derivatives of E as follows:

$$f=-\frac{dE}{dZ}=-\frac{9\gamma}{2Z_0}\left[\left(\frac{Z_0}{Z}\right)^4-\left(\frac{Z_0}{Z}\right)^{10}\right] \text{, (3)}$$

$$k_{vdW}=-\frac{d^2E}{dZ^2}=-\frac{27\gamma}{Z_0^2}\left[-\frac{2}{3}\left(\frac{Z_0}{Z}\right)^5+\frac{5}{3}\left(\frac{Z_0}{Z}\right)^{11}\right]. \quad (4)$$

The adhesion force (F) can then be obtained by:

$$F=\pi a^2 f, \quad (5)$$

where a is the contact radius at "jump-off" obtained using Carpick's solution[3] to the Maugis-Dugdale model as:

$$a=\left[1.54+0.279\left(\frac{2.28\mu^{1.3}-1}{2.28\mu^{1.3}+1}\right)\right]\left[\frac{0.98\left(1-e^{-1.082\mu}\right)}{1+0.98\left(1-e^{-1.082\mu}\right)}\right]^{2/3}\left(\frac{K}{\pi\gamma R^2}\right)^{-1/3}, \quad (6)$$

The parameter μ is defined as:

$$\mu=1.157\left(\frac{16R\gamma^2}{9K^2Z_0^3}\right)^{1/3}, \quad (7)$$

μ ranges between 0 and infinity, which correspond to the Derjaguin-Muller-Toporov (DMT) and Johnson-Kendall-Roberts (JKR) models, respectively. R is the tip radius, γ and Z_0 are defined in Eq. (2), and K is the reduced elastic modulus:

$$K=\frac{4}{3}\frac{1}{\left(1-v_1^2\right)/E_1+\left(1-v_2^2\right)/E_2}. \quad (8)$$

where E_1, v_1 and E_2, v_2 are the elastic moduli and the Poisson's ratios for the tip and specimen, respectively. Since the specimen thickness is low, the elastic properties of amorphous silicon dioxide (SiO_2) were used in the analysis. The elastic properties for SiO_2 were taken as E_{SiO_2}=70 GPa, v_{SiO_2}=0.3 (See supporting information in reference 2)[2]. The equilibrium separation of the surface Z_0 was set as 0.30 nm based on the interaction between graphene and SiO_2[4], same value was assumed for MXene as well. The maximum adhesive force can then be obtained at k_{vdW}=0. The maximum force gradients (F_k) for SiO_2, graphene, and MXene are 68750N m^{-1},

28640N m^{-1}, and 13624N m^{-1}, respectively, with $\pm$10% error. For comparison, the dashed straight lines with the slope of the cantilever's spring stiffness were plotted tangential to the curves at the maximum adhesive force points in Supplementary Figure 1. These results demonstrate that the measured adhesion is the maximum adhesion of the sample surfaces.

Supplementary Note 2: Determination of λ in Maugis-Dugdale Theory for this experiment

For Eq. (1) in the main text, λ is the coefficient ranging between 1.5 and 2. The JKR theory and DMT theory describe two extreme contacts between spherical particle and flat surface when λ=1.5, and λ=2, respectively.

An empirical fitting equation often used[3] to solve for the coefficient λ is employed:

$$\lambda = \left| -\frac{7}{4} + \frac{1}{4}\left(\frac{4.04\mu^{1.4}-1}{4.04\mu^{1.4}+1}\right) \right| . \quad (9)$$

where μ is defined in Eq. (7). The solved λ varies for each case, depending on the jump-off adhesion force. From the calculations, λ=1.613 for SiO_2, λ=1.587, 1.543, 1.543 for mono-, bi-, and tri-layer graphene, λ=1.560 and 1.558 for 1- and 15-monolayer $Ti_3C_2T_x$, and 1.602 and 1.602 for 1- and 19-monolayer Ti_2CT_x, respectively.

Supplementary Note 3: XPS analysis of $Ti_3C_2T_x$ and Ti_2CT_x MXene samples

We examined MXene surface chemistry by XPS. The commonly anticipated functional groups on MXene flakes are -OH, -O- and -F. XPS survey for $Ti_3C_2T_x$ MXene film shows F/O atomic ratio 0.37 (Supplementary Figure 4), while for Ti_2CT_x MXene film it is 0.32 (Supplementary Figure 4). Thus, the results indicate almost same F/O ratio for both MXenes (as expected). Hence, we conclude that surface chemistry is more or less same for both $Ti_3C_2T_x$ and Ti_2CT_x MXenes.

According to nuclear magnetic resonance studies[5], the majority of O atoms for MXene samples produced by MILD method belong to bridging Ti-O-Ti groups.

Supplementary Note 4: Statistical variation in measured adhesion energy

The measured adhesion energy variation among different flakes can be found in Figure 5a. For each type of graphene flake (mono-, bi-, or tri-layer), experiment numbers 1-27 are from batch 1. 1-9 represent measurements for #1 graphene flake, 10-18 for #2, and 19-27 for #3 graphene flake. Experiment numbers 28-54 are from batch 2. 28-36 represent measurements for #4 graphene flake, 37-45 for #5, and 46-54 for #6 graphene flake. For MXene flakes, experiment numbers 1-81 are from batch 1 (total of 9 different thickness). Experiment numbers 1-9 are for thickness #1, 10-18 for thickness #2, 19-27 for thickness #3, 28-36 for thickness #4 37-45 for thickness #5, 46-54 for thickness #6, 55-63 for thickness #7, 64-72 for thickness #8, 73-81 for thickness #9. Experiment numbers 82-90 and 91-99 are from batch 2 and 3 individually. Experiment numbers 100-180 are from batch 4 (total of 9 thicknesses) and the numbers have same meaning as for batch 1. Experiment numbers 181-189 and 190-199 are from batches 5 and 6 individually. The maximum fluctuations of measured adhesion energy over the corresponding average values for graphene samples are shown in Supplementary Table 1. The maximum fluctuations of measured adhesion energy over the corresponding average values for MXene samples are shown in Supplementary Table 2. The maximum fluctuation for adhesion energy measured for $Ti_3C_2T_x$ and Ti_2CT_x is within 12% of the average. Therefore, there is no thickness dependency observed.

All adhesion measurements were performed using the same AFM tip. The tip was found to be intact after each experiment under SEM and was calibrated before and after each experiment. For each number of layers of graphene (mono-, bi-, and tri- graphene samples), 6 flakes were chosen. 9 measurements were conducted on each flake. For each thickness of $Ti_3C_2T_x$ or Ti_2CT_x flake, 6

flakes were selected and 3 measurement were conducted on the grid areas as illustrated in the Supplementary Figure 9a.

Samples from three separate batches were tested. The batch-to-batch variations in measured adhesion energies are small: within 7% and 8% of the average values for $Ti_3C_2T_x$ and Ti_2CT_x, respectively. The details of these measurements and comparisons are provided in Supplementary Figure 7.

The calculation data on adhesion energies from all measurements are provided in the Supplementary data in Excel format.

Supplementary Table 1. Statistical variation of adhesion energy measurements for graphene

Adhesion Energy, J m^{-2}	Sample	Experiment Number		
		1-9 (batch 1, flake #1)	10-18 (batch 1, flake #2)	19-27 (batch 1, flake #3)
Maximum fluctuation/ Average value, %	Mono-layer	4.67	4.81	4.67
	Bi-layer	3.20	1.50	2.72
	Tri-layer	4.96	5.03	5.80
Adhesion Energy, J m^{-2}	Sample	28-36 (batch 2, flake #4)	37-45 (batch 2, flake #5)	46-54 (batch 2, flake #6)
Maximum fluctuation/ Average value, %	Mono-layer	5.09	5.99	4.67
	Bi-layer	4.55	4.68	4.30
	Tri-layer	2.00	3.03	3.29

Supplementary Table 2. Statistical variation of adhesion energy measurements for MXene

Adhesion Energy, J m^{-2}	Sample	Experiment Number				
		1-9 (batch 1, thickness #1)	10-18 (batch 1, thickness #2)	19-27 (batch 1, thickness #3)	28-36 (batch 1, thickness #4)	37-45 (batch 1, thickness #5)
Maximum fluctuation/ Average value, %	$Ti_3C_2T_x$	3.94	4.55	5.48	2.70	1.67
	Ti_2CT_x	7.68	9.47	6.15	4.68	8.97
Adhesion Energy, J m^{-2}	Sample	46-54 (batch 1, thickness #6)	55-63 (batch 1, thickness #7)	64-72 (batch 1, thickness #8)	73-81 (batch 1, thickness #9)	82-90 (batch 2)
Maximum fluctuation/ Average value, %	$Ti_3C_2T_x$	0.73	7.38	11.21	8.31	1.78
	Ti_2CT_x	9.47	5.46	11.68	9.15	6.00
Adhesion Energy, J m^{-2}	Sample	91-99 (batch 3)	100-108 (batch 4, thickness #1)	109-117 (batch 4, thickness #2)	118-126 (batch 4, thickness #3)	127-135 (batch 4, thickness #4)
Maximum fluctuation/ Average value, %	$Ti_3C_2T_x$	4.23	5.91	8.12	8.90	11.98
	Ti_2CT_x	6.21	7.68	9.47	6.15	4.68
Adhesion Energy, J m^{-2}	Sample	136-144 (batch 4, thickness #5)	145-153 (batch 4, thickness #6)	154-162 (batch 4, thickness #7)	163-171 (batch 4, thickness #8)	172-180 (batch 4, thickness #9)
Maximum fluctuation/ Average value, %	$Ti_3C_2T_x$	5.16	8.22	0.92	10.18	11.21
	Ti_2CT_x	8.97	9.47	5.46	11.13	9.15
Adhesion Energy, J m^{-2}	Sample	181-189 (batch 5)	190-198 (batch 6)			
Maximum fluctuation/ Average value, %	$Ti_3C_2T_x$	10.02	3.34			
	Ti_2CT_x	8.00	7.75			

Supplementary Figures

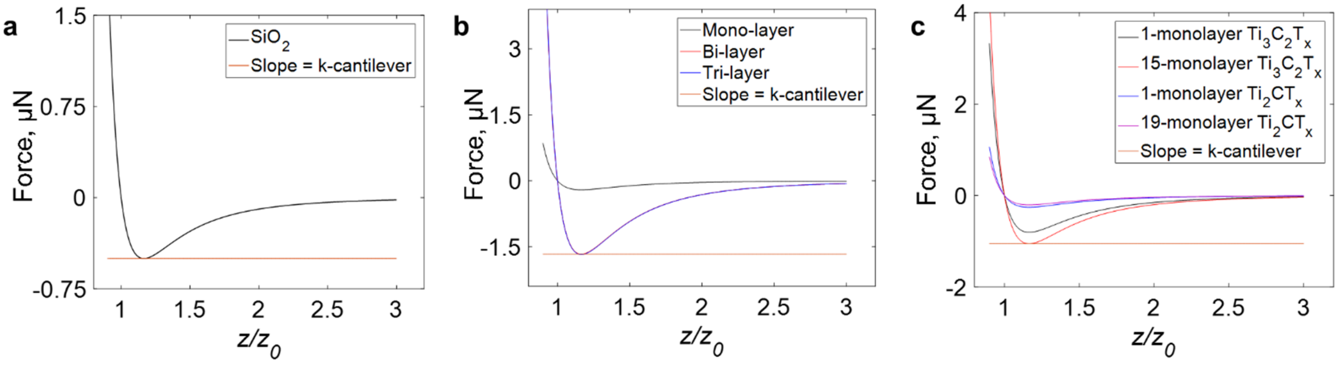

Supplementary Figure 1. Force curves for different samples used in this study. (a) SiO_2/SiO_2, (b) SiO_2/graphene and (c) SiO_2/MXene.

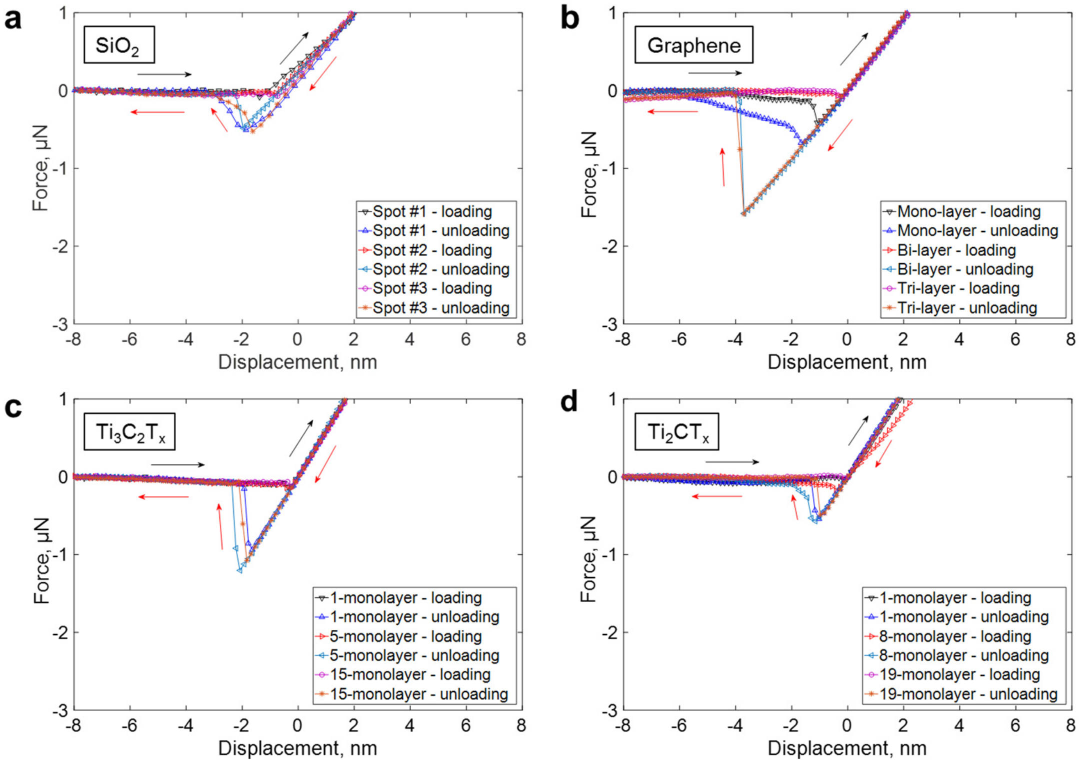

Supplementary Figure 2. Force *versus* displacement responses for different interfacial interactions. (a) SiO_2/SiO_2, (b) SiO_2/graphene, (c) $SiO_2/Ti_3C_2T_x$, (d) SiO_2/Ti_2CT_x.

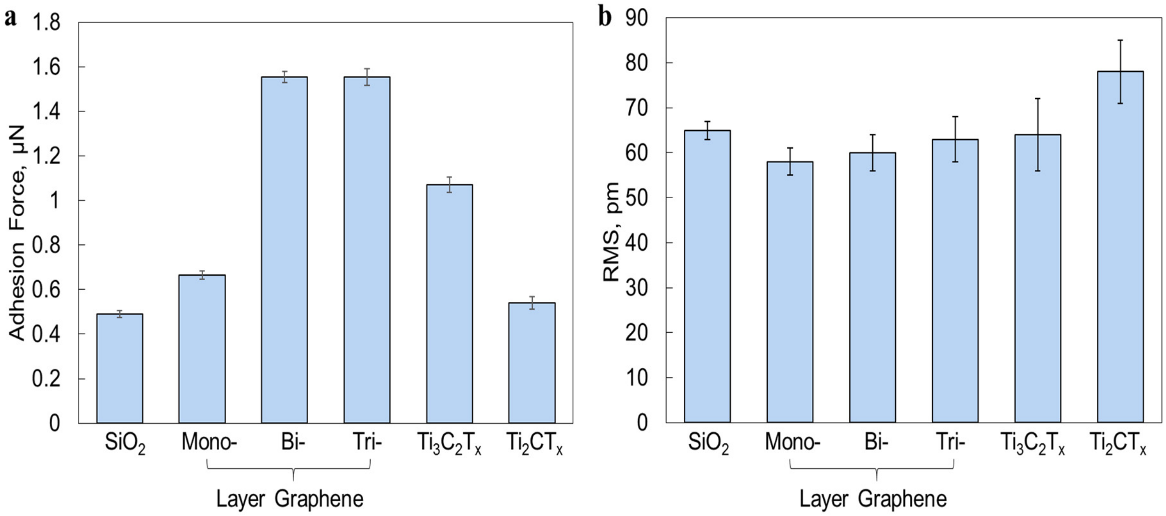

Supplementary Figure 3. Average measured (a) adhesion forces and (b) RMS values with error bars indicating standard deviations.

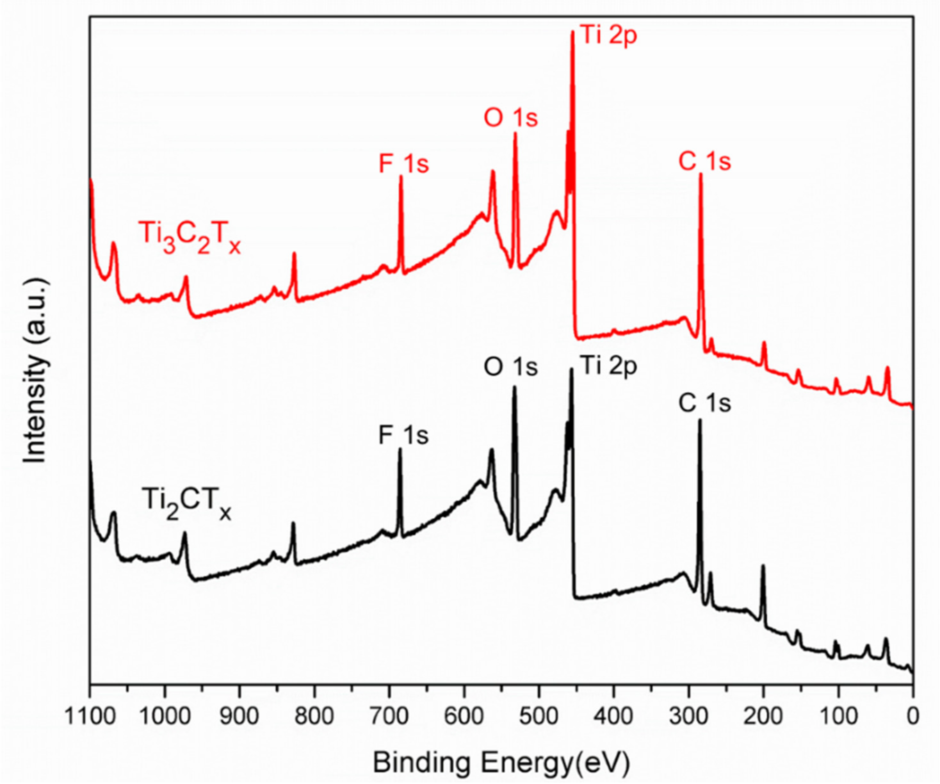

Supplementary Figure 4. XPS survey spectra of $Ti_3C_2T_x$ and Ti_2CT_x MXene films.

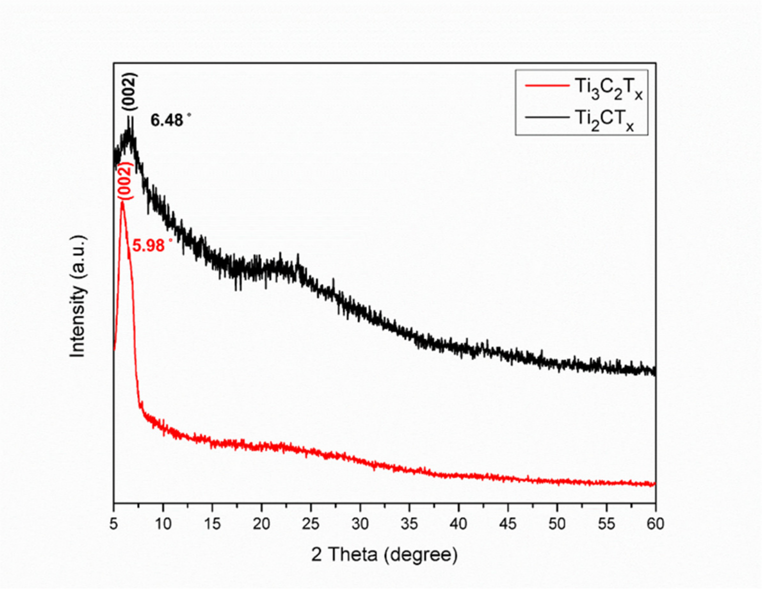

Supplementary Figure 5. XRD patterns of $Ti_3C_2T_x$ and Ti_2CT_x MXene thin films.

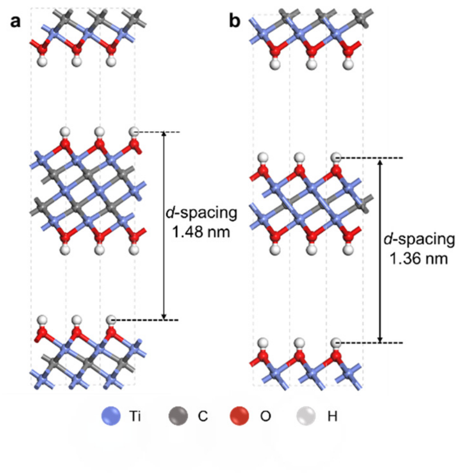

Supplementary Figure 6. Atomic structures of (a) $Ti_3C_2T_x$, (b) Ti_2CT_x MXenes and their corresponding d −spacing values calculated from XRD (Supplementary Figure 5). The number of MXene monolayers in a thin film was calculated as Number of monolayers$=\frac{\text{Film thickness (from AFM)}}{d\text{-spacing}}$.

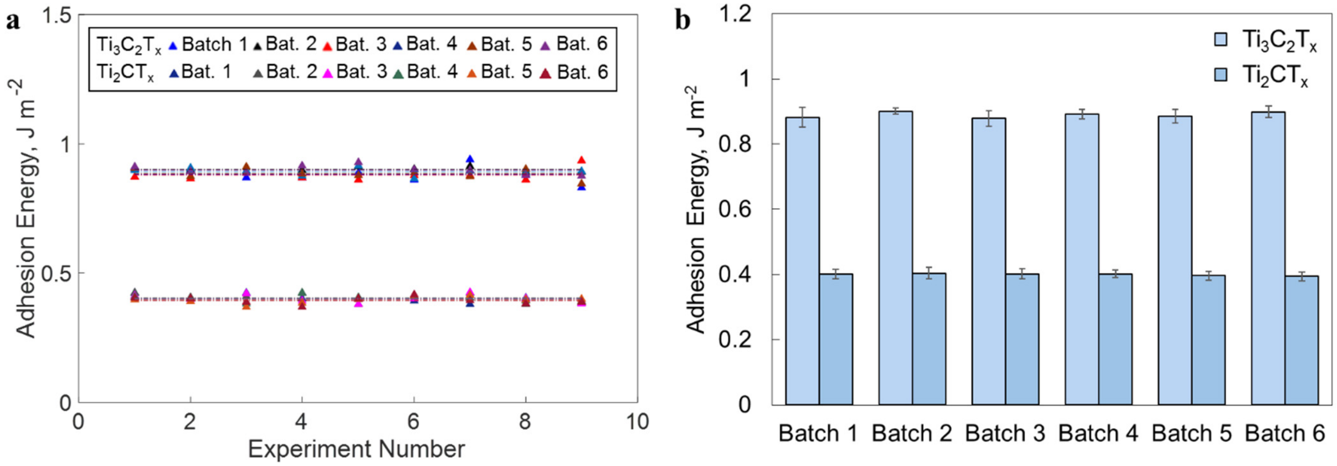

Supplementary Figure 7. (a) Batch-to-batch variations of adhesion energy, (b) average adhesion energies with error bars indicating standard deviations.

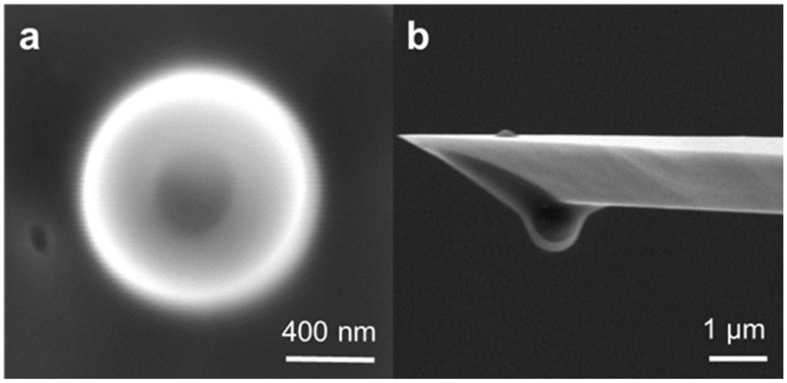

Supplementary Figure 8. SEM images of AFM SiO_2 microsphere tip. (a) Top and (b) side view of the microsphere tip attached to the cantilever.

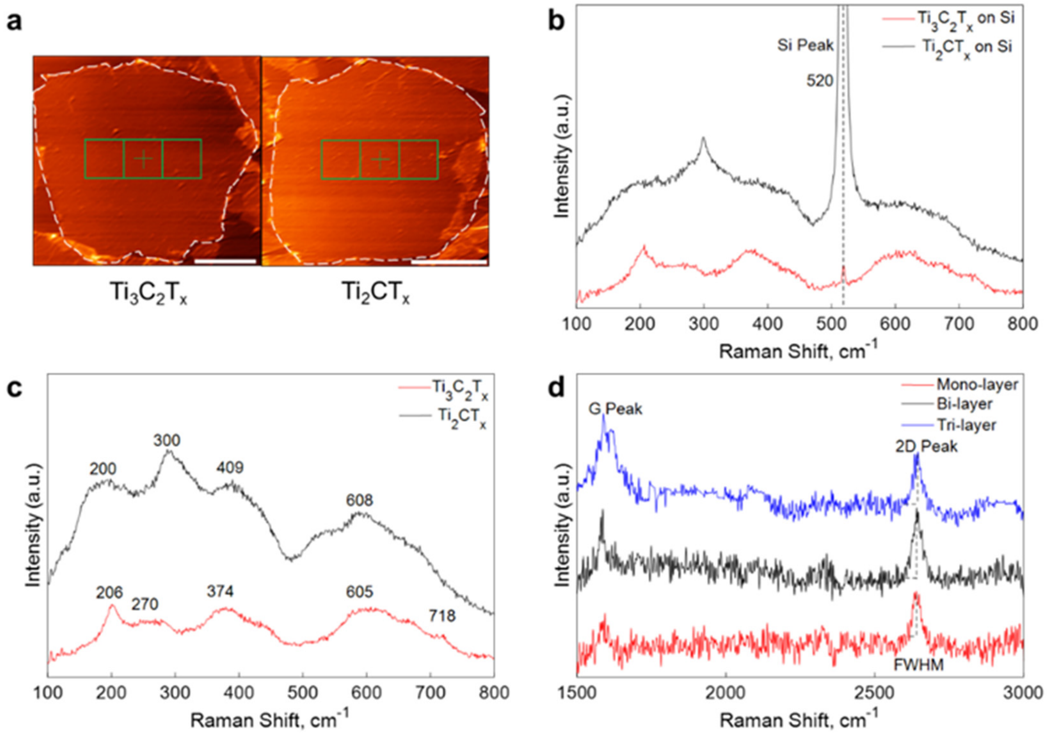

Supplementary Figure 9. (a) AFM scans of representative MXene flakes (scale bar 1 μm), which were divided into 3 scanning areas individually. Raman spectra of (b) $Ti_3C_2T_x$ and Ti_2CT_x on Si, (c) $Ti_3C_2T_x$, Ti_2CT_x on the cover glass, and (d) mono-, bi-, and tri-layer graphene.

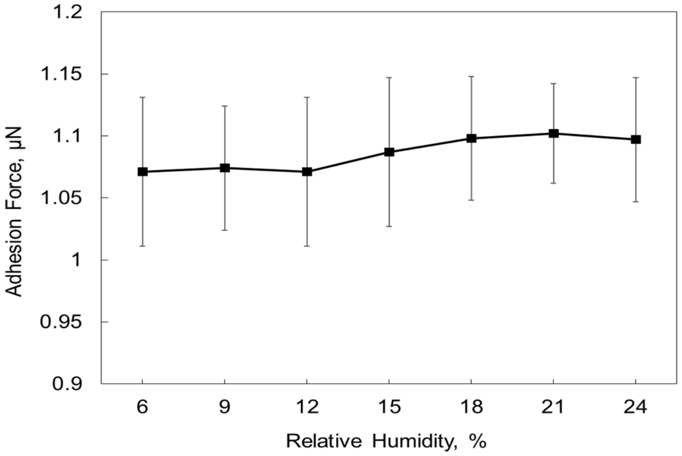

Supplementary Figure 10. Adhesion force of MXene film ($Ti_3C_2T_x$) *vs* relative humidity.

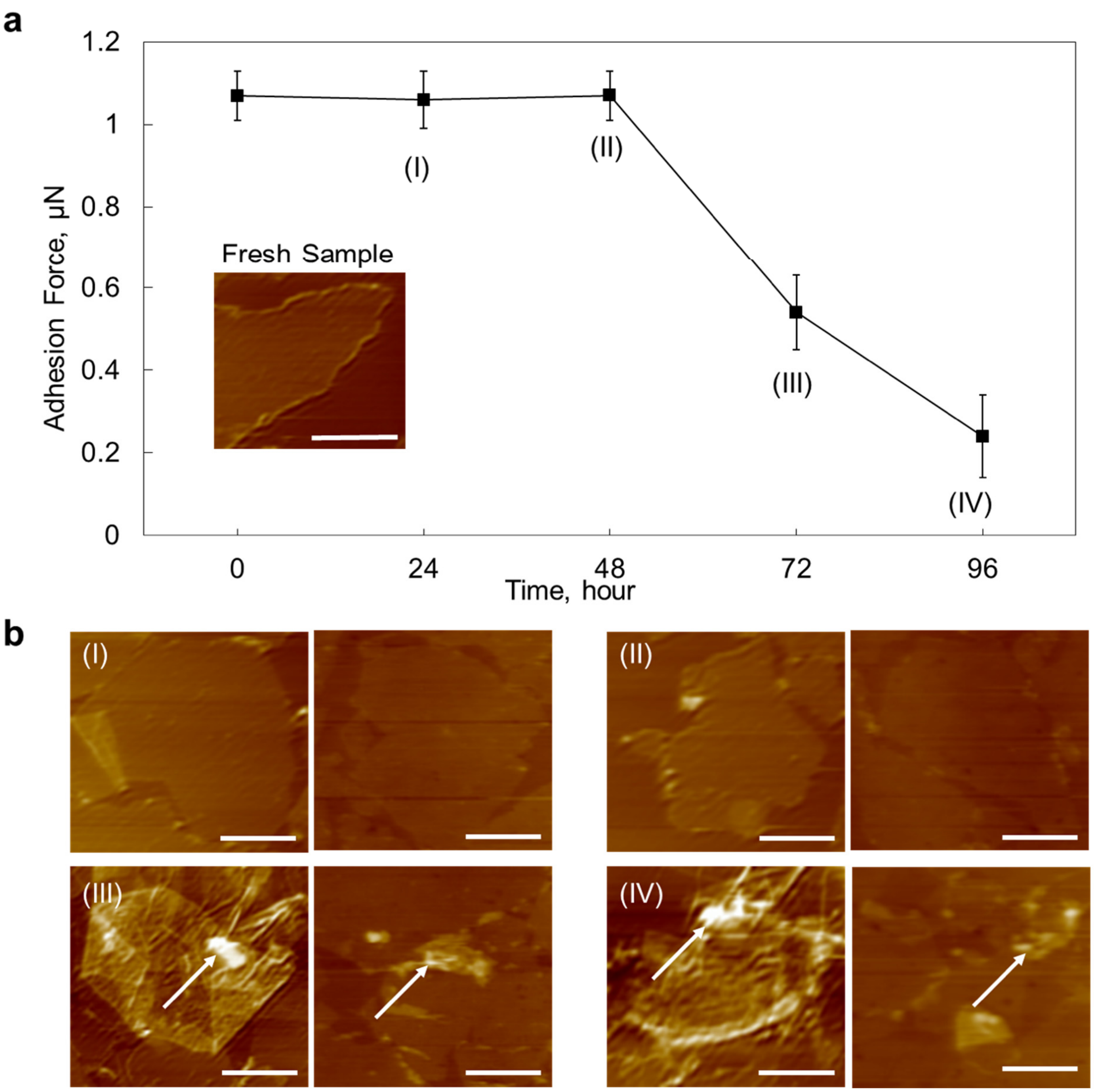

Supplementary Figure 11. (a) Adhesion force *vs* time of exposure in air under room temperature for $Ti_3C_2T_x$ flakes and (b) AFM images of $Ti_3C_2T_x$ flakes taken at the time points indicated by roman numbers that correspond to the points in panel (a). All scale bars are 1 µm. The pairs of AFM images show results from two different experiments, which were also averaged in panel (a). Arrows indicate TiO_2 nanoparticles formed as result of MXene oxidation.

Supplementary References

1. Cappella, B., & Dietler, G. Force-Distance Curves by Atomic Force Microscopy. *Surf. Sci. Rep*. **34**, 1-104 (1999).
2. Jiang, T. & Zhu, Y. Measuring Graphene Adhesion Using Atomic Force Microscopy with A Microsphere Tip. *Nanoscale* **7**, 10760-10766 (2015).
3. Carpick, R. W., Ogletree, D. F. & Salmeron, M. A General Equation for Fitting Contact Area and Friction *vs* Load Measurements. *J. Colloid Interface Sci.* **211**, 395-400 (1999).
4. Gao, W., Xiao, P., Henkelman, G., Liechti, K. M. & Huang, R. Interfacial Adhesion between Graphene and Silicon Dioxide by Density Functional Theory with Van Der Waals Corrections. *J. Phys. D: Appl. Phys.* **47**, 255301 (2014).
5. Hope, M. A. *et al.* NMR Reveals the Surface Functionalisation of Ti_3C_2 MXene. *Phys. Chem. Chem. Phys.* **18**, 5099-5102 (2016).
